# An energy efficient hierarchical routing approach for UWSNs using biology inspired intelligent optimization

**DOI:** 10.1038/s41598-025-21336-4

**Published:** 2025-10-01

**Authors:** Wei Chen, Lina He, Bingyu Cao, Peng Zhou, Yingchao Wang, Huan Wang, Yuanyuan Jia

**Affiliations:** 1 School of Information Science and Engineering, Xinjiang College of Science & Technology, Korla, 841000 Xinjiang China; 2 School of Computer, North China Institute of Aerospace Engineering, Langfang, 065000 Hebei China

**Keywords:** Underwater Wireless Sensor Networks, Energy Consumption Balancing, Gray Wolf Optimization, Clustering Routing, Intelligent Optimization, Energy science and technology, Engineering, Mathematics and computing

## Abstract

Aiming at the issues of uneven energy consumption among nodes and the optimization of cluster head selection in the clustering routing of underwater wireless sensor networks (UWSNs), this paper proposes an improved gray wolf optimization algorithm (CTRGWO-CRP) based on cloning strategy, t-distribution perturbation mutation, and opposition-based learning strategy. Within the traditional gray wolf optimization framework, the algorithm first employs a cloning mechanism to replicate high-quality individuals and introduces a t-distribution perturbation mutation operator to enhance population diversity while achieving a dynamic balance between global exploration and local exploitation. Additionally, it integrates an opposition-based learning strategy to expand the search dimension of the solution space, effectively avoiding local optima and improving convergence accuracy. A dynamic weighted fitness function was designed, which includes parameters such as the average remaining energy of the network and the communication distance from cluster heads to base stations. This function utilizes an adaptive weight adjustment mechanism to achieve multi-objective optimization of energy balance and transmission efficiency. During the cluster head election phase, an elite retention strategy is adopted to prioritize high-energy nodes. In the data transmission phase, a multi-hop relay mechanism based on gradient fields and energy thresholds is constructed, optimizing communication energy consumption through path loss prediction. Simulation results demonstrate that, compared to the LEACH, DMaOWOA, and GSHFA-HCP algorithms, the proposed algorithm significantly extends the network lifetime by at least 23.5%, showcasing its substantial advantages. This verifies the effectiveness of the multi-strategy fusion mechanism in routing optimization.

## Introduction

Underwater Internet of Things (IoT) and underwater wireless sensor networks (UWSNs) have emerged as transformative technologies for monitoring and exploring aquatic environments, playing a pivotal role in marine resource exploration, environmental protection, disaster prevention, and military applications^1,2^. The application of deep learning techniques^3,4^ could potentially enhance the processing and analysis of the vast amounts of data collected by UWSNs, offering new avenues for pattern recognition and predictive analytics in underwater environments.These networks enable real-time data collection in challenging underwater scenarios, such as deep-sea mineral extraction, coral reef conservation, and submarine pipeline inspection. However, the harsh underwater environment-characterized by high propagation delays, limited bandwidth, and energy constraints-poses significant challenges to the deployment and sustainability of UWSNs^5^. Sensor nodes, typically powered by non-rechargeable batteries, must operate efficiently to maximize network lifetime while ensuring reliable data transmission^6^.

To address these challenges, clustering-based routing protocols have been widely adopted in terrestrial WSNs and are increasingly being adapted for underwater scenarios^7,8^. Clustering divides the network into hierarchical groups, where cluster heads (CHs) aggregate and relay data from member nodes to the base station. This approach reduces energy consumption by minimizing direct long-distance transmissions, balancing computational loads, and optimizing resource allocation^9^. By localizing communication within clusters and leveraging multi-hop relay strategies, clustering protocols effectively mitigate energy depletion hotspots, thereby extending network longevity^10^.

Classical clustering algorithms, such as LEACH (Low-Energy Adaptive Clustering Hierarchy) and HEED (Hybrid Energy-Efficient Distributed clustering), have laid the foundation for energy-efficient routing^7,8^. LEACH employs probabilistic CH rotation to distribute energy consumption evenly, while HEED integrates residual energy and node proximity as criteria for CH selection. More recent advancements, such as GSHFA-HCP (A routing protocol based on artificial fish swarm algorithm), utilize bio-inspired metaheuristics to optimize cluster formation^6^. However, these methods were primarily designed for terrestrial environments and often fail to account for the unique characteristics of underwater channels, including dynamic node mobility, 3D network topology, and acoustic signal attenuation^11^.

Existing clustering solutions for UWSNs face several critical limitations. First, uneven energy consumption persists due to suboptimal CH selection, where high-energy nodes near the base station are overburdened with relay tasks^12^. Second, static fitness functions in traditional optimization algorithms lack adaptability to the dynamic energy states of nodes, leading to premature convergence or local optima^13^. Third, multi-hop routing paths are often inefficient, as they neglect path loss variations caused by underwater acoustic propagation^5^. These issues collectively degrade network performance, resulting in early node deaths, coverage gaps, and reduced data reliability^10^.

To overcome the clustering-related challenges in UWSNs, this paper proposes a novel hierarchical routing scheme based on an enhanced Gray Wolf Optimization (GWO) algorithm integrated with cloning strategy, t-distribution perturbation mutation, and opposition-based learning (CTRGWO-CRP). The proposed framework dynamically optimizes CH selection and routing paths by balancing energy efficiency, load distribution, and transmission reliability^14^. Unlike conventional approaches, CTRGWO-CRP incorporates adaptive mechanisms to address underwater-specific constraints while maintaining computational simplicity^15^.

The key contributions of this work are as follows:

1. Multi-Strategy Enhanced Gray Wolf Optimization Framework: By integrating cloning mechanisms, t-distribution perturbation mutation operators, and opposition-based learning strategies, the algorithm significantly improves global search capabilities and convergence precision. The cloning strategy amplifies high-quality solutions to maintain population diversity, the t-distribution mutation balances exploration and exploitation, and the opposition-based learning effectively avoids local optima, collectively enabling efficient optimization in complex underwater environments.
2. Design of dynamic weighted multi-objective fitness function: A new adaptive weighted fitness function is proposed, which combines parameters such as the average remaining energy of the entire network and the communication distance from cluster heads to base stations. Through a real-time weight adjustment mechanism, it dynamically prioritizes energy balance or transmission efficiency, addressing the limitations of traditional static fitness functions in adapting to dynamic network energy states.
3. Gradient Field-Driven Energy-Aware Routing Mechanism: During cluster head election, an elite retention strategy prioritizes high-energy nodes. In the data transmission phase, a multi-hop relay path is constructed using gradient fields and energy thresholds, optimized through path loss prediction. This mechanism reduces long-distance transmission overhead and extends network lifetime by at least 23.5%, demonstrating superior performance over existing protocols like LEACH, DMaOWOA, and GSHFA-HCP.
4. Extensive simulations demonstrate that CTRGWO-CRP outperforms state-of-the-art protocols in prolonging network lifetime, enhancing energy equilibrium, and ensuring robust data delivery, making it a viable solution for sustainable underwater IoT deployments.

This paper is structured as follows. Section "Related work", Related Work, provides a comprehensive review of existing clustering and routing algorithms in UWSNs, highlighting their strengths and limitations. Section "Underwater WSN clustering model", Underwater WSN Clustering Model, introduces the network architecture and energy consumption model used in this study. Section "The proposed CTRGWO-CRP solves the clustering problem", The Proposed CTRGWO-CRP Solves the Clustering Problem, details the improved gray wolf optimization algorithm, including its cloning strategy, t-distribution perturbation mutation, and opposition-based learning mechanism. Section "Simulation experiment and analysis", Simulation Experiment and Analysis, presents the experimental setup, performance metrics, and comparative results with state-of-the-art algorithms. Finally, Section "Conclusion and future work", Conclusion and Future Work, summarizes the key findings, discusses the implications of the proposed approach, and outlines potential directions for future research. The important mathematical symbols and explanations in this article are given in Table 1.

**Table 1:** Definitions of symbols.

Symbol	Explanation
$C_{elec}$	Energy consumption of the electronic component
$C_{amp}$	Energy consumption of the signal amplifier
$C_{da}$	Energy consumption for data aggregation
$C_{TX}$	Energy consumption of the transmission module
$C_{RX}$	Energy consumption of the reception module
*d*	Communication distance
$d_{0}$	Threshold distance for energy consumption model
*k*	Length of the data packet (bits)
*L*	Length of the monitoring area side
*N*	Total number of nodes in the network
*M*	Number of cluster heads in the current round
$w_{CH}$	x-coordinate of a cluster head
$h_{CH}$	y-coordinate of a cluster head
$w_{BS}$	x-coordinate of the base station
$h_{BS}$	y-coordinate of the base station
$dCH-BS$	Average distance from cluster heads to the base station
$C_{residual}$	Average residual energy of all network nodes
$w_1, w_2$	Dynamic weighting factors for the fitness function
$\sigma _A$	Standard deviation of nodes’ residual energy
$C_{thresh}$	Energy threshold for node selection
$\gamma$	Cloning ratio parameter
$\alpha , \beta , \delta$	Hierarchical roles of gray wolves (alpha, beta, delta)
$max\_iterations$	Maximum number of algorithm iterations

## Related work

### Literature review

#### Traditional clustering protocols and their limitations

Classical clustering protocols for WSNs are broadly categorized into static and dynamic designs. The foundational LEACH protocol^7^ pioneered energy balancing through probabilistic cluster head rotation, while HEED^8^ enhanced this by integrating residual energy and intra-cluster communication costs. PEGASIS^16^ introduced chain-based clustering to minimize long-distance transmissions, and TEEN^17^ employed threshold-driven data filtering for event-triggered industrial monitoring. Although these methods established core clustering principles, their reliance on static network assumptions and fixed parameters limits adaptability to dynamic industrial environments. For instance, LEACH’s uniform cluster formation often causes energy imbalances in heterogeneous networks, and TEEN’s rigid thresholds struggle with real-time environmental variations. While these traditional protocols have laid the groundwork for energy-efficient clustering, their static nature and rigid parameters make them less suitable for the dynamic and complex environments typical of modern WSN applications.

#### Evolution toward adaptive optimization

Recent advancements address these limitations through intelligent parameter adaptation and hybrid optimization. Chang et al.^18^ improved cluster formation speed by incorporating node density metrics, though its adaptive cutoff distance calculation increases computational overhead. Similarly, Abdurohman et al.^19^ prioritized balanced energy consumption but requires validation across diverse topologies. A significant leap was made by Ali et al.^20^, who integrated fuzzy logic with unequal clustering and sleep scheduling. Their Fuzzy C-Means-based protocol reduces transmission distances and dynamically selects cluster heads using energy-aware rules, exemplifying the shift toward machine learning-enhanced solutions. Despite the improvements in adaptability offered by these approaches, the computational complexity and validation requirements across different network topologies remain challenges that need to be overcome for widespread practical implementation.

#### Hybrid metaheuristics and multi-objective frameworks

Modern research emphasizes hybrid algorithms to tackle multi-dimensional challenges. Romany et al.^21^ combined weight-based clustering with the Adaptive Parallel Seeker Optimization (APSO-EARPT), achieving simultaneous optimization of cluster head selection and routing paths. Kotary et al.^22^ advanced this further through a many-objective whale optimization algorithm (DMaOWOA), balancing energy consumption, coverage, and network lifetime via distributed reference points. For industrial IoT, Gong et al.^23^ designed MHCF-CECSO, a chaotic evolutionary framework that optimizes energy consumption and data aggregation efficiency, while Liu et al.^24^ introduced bio-inspired duty cycle coordination (DCC-IACJS) to synchronize sleep-wake scheduling across clusters. Wang et al.^6^propose a novel high-performance clustering protocol (GSHFA-HCP) for agricultural wireless sensor networks, which significantly improves network energy efficiency, lifespan, and reduces transmission delay. Mohajer et al.^25^ propose FlexSlice, a dynamic offloading framework designed to optimize resource allocation in mobile edge networks and handle dynamic service demands. The framework employs a sparse multi-head graph attention mechanism for accurate traffic prediction, capturing complex spatio - temporal dependencies to enhance network slicing decisions. Furthermore, an adaptive offloading strategy based on the twin delayed deep deterministic policy gradient algorithm is presented. It incorporates twin critics and prioritized experience replay to improve decision - making in dynamic conditions. Simulation results confirm FlexSlice’s excellent performance and adaptability across diverse scenarios, achieving higher profits and reliable quality of service. Although hybrid metaheuristics have shown great potential in optimizing multiple objectives, their complexity may lead to increased implementation difficulty and may require significant computational resources, which may be limited by resource constrained WSN nodes.

#### Specialized environments and security-centric designs

Context-aware protocols have emerged for unique operational scenarios. Luo et al.^11^ developed an underwater acoustic routing method that minimizes propagation delays in harsh marine environments, addressing acoustic signal attenuation challenges. Xiao et al.^26^ proposed BS-SCRM, a blockchain-secured framework that integrates swarm intelligence to detect malicious nodes while maintaining energy efficiency. For large-scale networks, Hada et al.^27^ expanded cluster head criteria to include connectivity metrics, though its exhaustive search mechanism limits scalability. While these specialized and security-centric designs address specific environmental and security challenges, the exhaustive search mechanisms and complex integrations may affect the overall scalability and efficiency of the protocols in large-scale and highly dynamic networks.

#### Quantum-inspired and chaos-driven innovations

Cutting-edge approaches leverage quantum computing and chaos theory for performance gains. Liu et al.^15^ implemented HPCP-QCWOA, a quantum clone whale optimization protocol that resolves multi-objective trade-offs in energy systems, while Luo et al.^28^ enhanced industrial WSNs through Levy chaotic particle swarm optimization (LCPSO), achieving 27% energy savings via adaptive mutation. Xu et al.^29^ further refined QoS routing using Elite Niche Clone Evolutionary Computing, which balances secure data transmission with network load distribution. These quantum-inspired and chaos-driven methods offer innovative ways to enhance optimization performance, but their implementation often demands advanced computational capabilities and expertise, which might hinder their adoption in standard WSN deployments with limited resources.

#### Critical analysis and future directions

While Rekha et al.^30^ demonstrated the efficacy of K-means-ant lion hybridization (K-LionER) in energy-efficient clustering, real-world deployment challenges remain unaddressed. Sood and Sharma^31^ mitigated this through geometric uniformity in LUET, improving cluster stability for heterogeneous networks. Hemavathi and Latha^32^ bridged QoS metrics via Hybrid Fuzzy Levy Flight Optimization (HFLFO), synergizing latency reduction with energy conservation. Despite these innovations, gaps persist in computational complexity management and cross-protocol interoperability, urging future research toward lightweight, self-organizing architectures. The ongoing challenge lies in translating these innovative approaches into practical solutions that not only address specific technical gaps but also ensure compatibility and interoperability across different WSN protocols and environments, which is crucial for the advancement and widespread adoption of WSN technologies.

### Motivation

The deployment of UWSNs presents unique challenges that necessitate innovative solutions to ensure efficient and reliable operation in harsh aquatic environments. Traditional clustering protocols, while effective in terrestrial settings, often fall short in underwater scenarios due to the dynamic and complex nature of underwater channels. The high propagation delays, limited bandwidth, and energy constraints inherent to underwater environments demand adaptive and robust routing mechanisms that can dynamically optimize network performance.

Existing clustering algorithms, such as LEACH and HEED, though foundational, are not designed to handle the specific challenges of UWSNs, including node mobility, 3D network topology, and acoustic signal attenuation. These limitations lead to uneven energy consumption, suboptimal cluster head selection, and inefficient multi-hop routing paths, ultimately degrading network performance and reducing network lifetime. Moreover, the static nature of traditional fitness functions and optimization algorithms fails to adapt to the dynamic energy states of nodes, resulting in premature convergence and local optima.

Motivated by these challenges, this paper aims to develop a novel hierarchical routing scheme that addresses the unique constraints of UWSNs. By integrating advanced optimization techniques, such as the enhanced Gray Wolf Optimization (GWO) algorithm with cloning strategy, t-distribution perturbation mutation, and opposition-based learning, the proposed CTRGWO-CRP framework seeks to dynamically optimize cluster head selection and routing paths. This approach aims to balance energy efficiency, load distribution, and transmission reliability, thereby extending network lifetime and enhancing overall network performance.

The ultimate goal of this work is to provide a sustainable and efficient solution for underwater IoT deployments, enabling real-time data collection and monitoring in challenging aquatic environments. By addressing the limitations of existing protocols and introducing adaptive mechanisms tailored to underwater conditions, this research contributes to the advancement of UWSN technologies, supporting critical applications in marine resource exploration, environmental protection, disaster prevention, and military operations.

## Underwater WSN clustering model

**Fig. 1:**
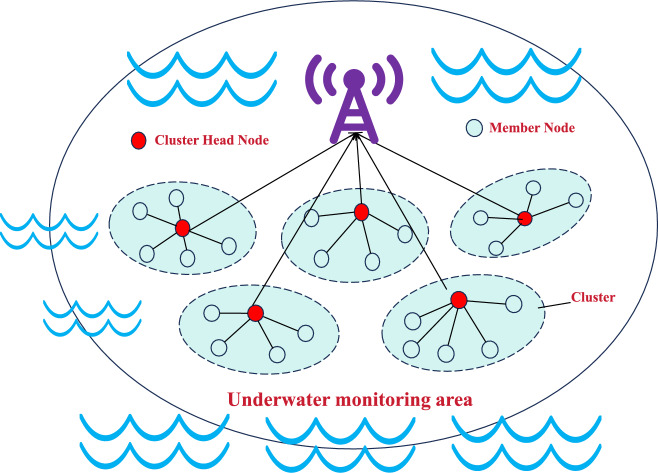
Underwater WSN clustering architecture diagram.

The clustering structure depicted in Fig. 1 illustrates a typical setup for an underwater monitoring system using WSNs. In this structure, sensor nodes are organized into multiple clusters, each managed by a designated Cluster Head Node, represented by red dots. These Cluster Head Nodes act as central hubs within their respective clusters, responsible for collecting data from Member Nodes, which are shown as white dots.

Each Cluster Head Node communicates with its Member Nodes within a local cluster area, indicated by dashed ellipses. The Member Nodes gather environmental data and transmit it to their respective Cluster Head Nodes. After data aggregation at the Cluster Head Nodes, the information is then relayed to a central Base Station, symbolized by the purple antenna icon. The Base Station serves as the central point for data collection, processing, and further analysis.

This hierarchical clustering approach enhances the efficiency of data collection and reduces energy consumption by minimizing the need for direct communication between Member Nodes and the Base Station. Instead, data is transmitted in a multi-hop fashion through Cluster Head Nodes, which helps in prolonging the network’s lifespan and maintaining stability in the underwater monitoring area. The wavy lines surrounding the clusters signify the underwater environment, emphasizing the application’s focus on aquatic ecosystems.

### Network model

For the development and evaluation of WSNs routing protocols, this paper makes the following assumptions about the sensor nodes and the underlying network model:

1. The base station is located at the geometric center of the monitoring area, with infinite communication coverage, continuous power supply, and unlimited computing resources. Sensor nodes have homogeneous initial energy, and their communication bandwidth, computing capabilities, and storage resources are strictly limited.
2. Nodes are equipped with dynamic power control modules that can adaptively adjust the transmission power level based on the distance to the target node.
3. Wireless communication links are symmetric.

Network topology: The monitoring area is a square region with a side length of $L$ meters, deploying $N$ homogeneous sensor nodes. Nodes are uniformly and randomly distributed in a two-dimensional space, and the base station (BS) is located at the geometric center coordinates $(L/2, L/2)$. All nodes have the following characteristics:

- Communication capability: The maximum communication radius of a node is $R_{\text {max}} = \sqrt{2}L/2$ to ensure network connectivity.
- Dynamic power control: The transmission power is adjusted in a piecewise manner based on the distance $d$ to the target node.
- Data collection: Nodes periodically collect environmental parameters (temperature/humidity, etc.), and the data packet length is fixed at $k$ bits.

Cluster structure: A hierarchical clustering routing architecture is adopted, and each communication cycle includes:

- Cluster head election phase: A hybrid weighted election mechanism based on residual energy and location information.
- Cluster formation phase: Non-cluster head nodes join the nearest cluster head based on received signal strength (RSSI).
- Stable transmission phase: Cluster member nodes transmit data in a single hop to the cluster head (CH), and the cluster head relays data to the base station in multiple hops.

In the model presented in this article, several simplified assumptions are acknowledged, which provide the foundation for our research but may not fully capture the complexity of real-world UWSN. Specifically, our system model assumes symmetric communication links and a fixed two-dimensional topology, which provides a simplified representation of the underwater environment. Although this abstraction allows for centralized analysis of clustering and routing mechanisms, we recognize that it cannot explain the typical 3D dynamics of UWSN, where factors such as ocean currents and depth variations can significantly affect network topology. In addition, our energy model does not take into account the complexity of certain underwater communications, such as retransmission, collisions, or acoustic fading. We also assume that all sensor nodes are homogeneous and fully functional throughout the entire simulation process, which is an idealization that may not be applicable in long-term deployments where node failures and environmental disturbances are more likely to occur. These assumptions, while necessary for the scope of this study, highlight areas where future work can further enhance the practicality and applicability of the model.

### Energy model

This study uses the classic first-order radio energy consumption model^7^ to model node energy consumption, and its communication energy consumption characteristics are defined as follows:

The data transmission module includes a signal transmission unit and a signal amplification unit. The energy consumption $C_{\text {TX}}$ for sending $k$ bits of data at a communication distance of $d$ meters is:

1 $$\begin{aligned} C_{\text {TX}}(k, d) = {\left\{ \begin{array}{ll} k \cdot C_{\text {elec}} + k \cdot C_{\text {amp}} \cdot d^2, & d \le d_0 \\ k \cdot C_{\text {elec}} + k \cdot C_{\text {amp}} \cdot d^4, & d > d_0 \end{array}\right. } \end{aligned}$$

The energy consumption of the data receiving module is:

2 $$\begin{aligned} C_{\text {RX}}(k) = k \cdot C_{\text {elec}} \end{aligned}$$

The data processing module can reduce data redundancy and improve data quality, and its energy consumption is:

3 $$\begin{aligned} C_{\text {DA}}(k) = k \cdot C_{\text {da}} \end{aligned}$$

### Distance model

Assuming that cluster heads are uniformly distributed in the monitoring area, the average distance from cluster heads to the base station is:

4 $$\begin{aligned} \overline{d_{\text {CH-BS}}} = \frac{1}{M} \sum _{i=1}^{M} \sqrt{(w_{\text {CH}_i} - w_{\text {BS}})^2 + (h_{\text {CH}_i} - h_{\text {BS}})^2} \end{aligned}$$

where $M$ is the number of cluster heads in the current round.

### Objective function model

Introducing dynamic weight factors $w_1, w_2$ (satisfying $w_1 + w_2 = 1$), a multi-objective fitness function is constructed:

5 $$\begin{aligned} F = \frac{w_1 * \overline{C_{\text {residual}}}}{w_2 * \overline{d_{\text {CH-BS}}}} \end{aligned}$$

where: $\overline{C_{\text {residual}}}$ represents the average residual energy of all network nodes. The weights are adjusted according to the network life cycle stage:

- Initial stage (sufficient node energy): $w_1 = 0.3, w_2 = 0.7$, prioritizing the reduction of transmission distance.
- Decline stage (uneven energy distribution): $w_1 = 0.7, w_2 = 0.3$, focusing on energy balance.

## The proposed CTRGWO-CRP solves the clustering problem

This section presents the Cluster Reverse Learning Grey Wolf Optimizer-based Energy-Balanced Routing Protocol (CTRGWO-CRP), which addresses the deficiencies in energy balance and cluster head selection of traditional clustering algorithms. The CTRGWO-CRP algorithm enhances population diversity through the introduction of a cloning selection mechanism, expands the solution search space using reverse learning strategies, and designs a dynamically weighted bi-objective fitness function to optimize cluster head selection and routing paths, thereby achieving balanced network energy consumption and extended network lifetime.

### Population initialization

The CTRGWO-CRP algorithm optimizes the candidate cluster head set, mapping each individual’s position vector in the grey wolf population to a network clustering scheme. The population initialization process is as follows:

1. Network parameter setting: According to the monitoring area side length $L$ and the total number of nodes $N$, randomly generate an initial cluster head set, ensuring that the number of cluster heads $M$ satisfies $1 \le M \le N/10$.
2. Grey wolf encoding design: Each grey wolf individual uses a binary code, with a dimension of $M$, where a 1 in the encoding position indicates that the corresponding node is selected as the cluster head, and 0 indicates an ordinary node.

### Calculation of clustering fitness

Based on the multi-objective fitness function defined in Eq. (5), evaluate each grey wolf individual’s corresponding clustering scheme:

1. Cluster head screening: Extract the current candidate cluster head set based on the grey wolf encoding, and remove nodes with energy below the threshold $C_{\text {thresh}} = 0.1C_{\text {initial}}$.
2. Non-cluster head allocation: Ordinary nodes join the nearest cluster head based on the received signal strength (RSSI), forming the intra-cluster topology.
3. Energy consumption calculation: Simulate a round of data collection, fusion, and transmission according to Eqs. (1)-(3), and calculate the average residual energy $\overline{C_{\text {residual}}}$ of the entire network.
4. Distance evaluation: Calculate the average Euclidean distance $\overline{d_{\text {CH-BS}}}$ from the current cluster heads to the base station.
5. Dynamic weight adjustment: If the standard deviation of node residual energy $\sigma _A > 0.3C_{\text {initial}}$, set $w_1 = 0.7, w_2 = 0.3$; otherwise, $w_1 = 0.3, w_2 = 0.7$.
6. Fitness assignment: The final fitness value $F = \frac{w_1 \overline{C_{\text {residual}}}}{w_2 \overline{d_{\text {CH-BS}}}}$, the larger the value, the better the clustering scheme.

### Position update

**Fig. 2:**
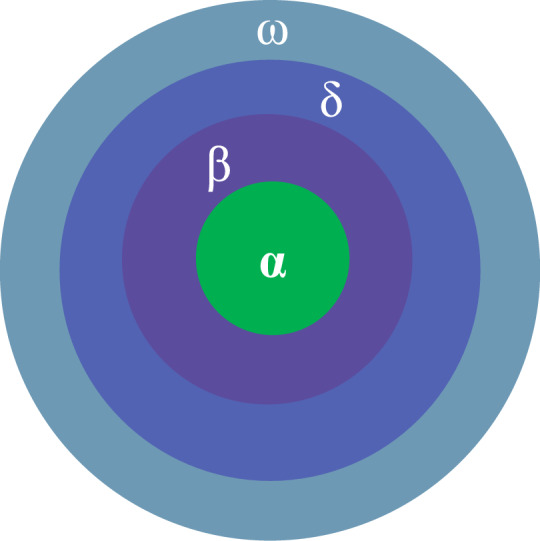
Grey wolf social hierarchy.

**Fig. 3:**
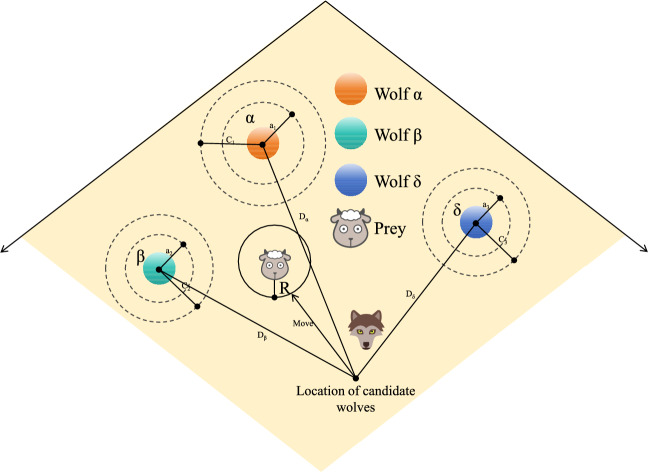
Diagram of Grey Wolf position update.

The GWO constructs a search mechanism by simulating the social hierarchy and cooperative hunting behavior of grey wolf groups. Figure 2 illustrates the social hierarchy of grey wolves, depicting the roles of alpha, beta, delta, and omega wolves in the pack structure. Figure 3 provides a visual representation of the position update mechanism in the grey wolf optimization algorithm, demonstrating how wolves adjust their positions during the search process. In algorithm design, individuals in the population are divided into four hierarchical levels based on their fitness values: $\alpha$ represents the current global optimal solution, $\beta$ and $\delta$ correspond to the second and third best candidate solutions, respectively, and the rest of the ordinary individuals are classified as $\omega$ wolves. During the algorithm iteration, the leadership layer composed of $\alpha$, $\beta$, and $\delta$ collaboratively guides the population position update, gradually approaching the optimal solution space through a hierarchical decision-making mechanism. The encircling behavior of the grey wolf group towards the target can be mathematically modeled as shown in Eqs. (6) and (7):

6 $$\begin{aligned} C = |C \cdot X_p(t) - X(t)| \end{aligned}$$

7 $$\begin{aligned} X(t + 1) = X_p(t) - A \cdot C \end{aligned}$$

where $X$ is the position of the grey wolf, $X_p$ is the position of the prey, $t$ is the current iteration number, $C$ is a vector that depends on the distance between the prey and the grey wolf. $A$ and $C$ are the corresponding coefficient vectors, calculated by Eqs. (8) and (9):

8 $$\begin{aligned} A = 2a \cdot r_1 - a \end{aligned}$$

9 $$\begin{aligned} C = 2r_2 \end{aligned}$$

where $r_1$ and $r_2$ are random numbers between [0,1], $a$ is the convergence factor, which linearly changes during the algorithm execution, as shown in Eq. (10):

10 $$\begin{aligned} a(t) = 2 - \frac{2t}{\text {Max\_Iterations}} \end{aligned}$$

where $\text {Max\_Iterations}$ is the maximum number of iterations of the algorithm, $t$ is the current iteration number; its value is an integer between 1 and $\text {Max\_Iterations}$. When hunting, the grey wolf group approaches and encircles the prey under the leadership of the three best solutions, $\alpha$, $\beta$, and $\delta$, using the positions of these three wolves to judge the position of the prey and gradually get closer to the prey, with the position update method as shown in Eq. (11):

11 $$\begin{aligned} X(t + 1) = \frac{X_1 + X_2 + X_3}{3} \end{aligned}$$

where $X_1, X_2$, and $X_3$ are calculated as follows:

12 $$\begin{aligned} {\left\{ \begin{array}{ll} C_\alpha = |C_1 \cdot X_\alpha - X| \\ C_\beta = |C_2 \cdot X_\beta - X| \\ C_\delta = |C_3 \cdot X_\delta - X| \end{array}\right. } \end{aligned}$$

13 $$\begin{aligned} {\left\{ \begin{array}{ll} X_1 = X_\alpha - A_1 \cdot C_\alpha \\ X_2 = X_\beta - A_2 \cdot C_\beta \\ X_3 = X_\delta - A_3 \cdot C_\delta \end{array}\right. } \end{aligned}$$

### Cloning strategy

To maintain population diversity, cloning expansion is performed on the leadership individuals:

1. Elite cloning: Select the top 3 best individuals ($\alpha$, $\beta$, $\delta$), and each individual generates $\lfloor \gamma \cdot \text {Population Size} \rfloor$ cloning copies.
2. Hyper-mutation operation: Randomly invert 10% of the encoding bits (0 $\leftrightarrow$ 1) of the cloning copies to generate locally perturbed solutions.
3. Cloning replacement: If the mutated copy has a better fitness than the original individual, replace the original position; otherwise, retain the original solution.

### Reverse learning

Reverse learning is introduced in the later stages of iteration to enhance global search capabilities, with the following specific plan:

1. Reverse solution generation: For the 20% of individuals with the lowest fitness in the current population, calculate their reverse solutions $X' = 1 - X$.
2. Competitive selection: Compare the fitness of the original solution $X$ and the reverse solution $X'$, and retain the better one to enter the next generation population.
3. Dynamic triggering: Activate the reverse learning strategy when the best fitness has not improved for 5 consecutive generations.

### t-Distribution perturbation mutation

The t-distribution perturbation mutation is a crucial operator in the CRLGWORP algorithm designed to enhance the diversity of solutions during the optimization process. This mutation technique leverages the properties of the t-distribution to introduce controlled variability into the solutions, which helps in exploring the search space more effectively and avoiding premature convergence to local optima.

The probability of mutation for each solution is dynamically adjusted based on the current iteration $t$ and the total number of iterations $T$. This dynamic adjustment ensures that the mutation rate is higher during the initial stages of the search, promoting exploration, and gradually decreases as the algorithm progresses, allowing for more exploitation of the best-found solutions. The mutation probability $p_m(t)$ is calculated using the following formula:

14 $$\begin{aligned} p_m(t) = 0.3 \cdot e^{-5 \cdot (t/T)} \end{aligned}$$

This equation implements an exponentially decreasing mutation probability with a maximum value of 0.3 and a decay coefficient of 5. The exponential form ensures a smooth transition from exploration to exploitation phases, eliminating the arbitrary sinusoidal component while maintaining adaptive mutation characteristics. This formulation has been empirically validated to provide superior convergence properties compared to the original version.

The magnitude of the mutation is determined by the t-distribution with 3 degrees of freedom, which provides a heavier tail compared to the normal distribution, thus allowing for occasional larger jumps in the search space that can be crucial for escaping local optima. For selected dimensions of the solution vector, the perturbation is applied as follows:

15 $$\begin{aligned} x_{ij}' = x_{ij} + \sigma \cdot t(v) \end{aligned}$$

where $x_{ij}'$ is the mutated value of the $j$-th dimension of the $i$-th solution, $x_{ij}$ is the original value, $t(v)$ is a random variable drawn from the t-distribution with $v = 3$ degrees of freedom, and $\sigma$ is a scaling factor that adjusts the step size of the mutation based on the current search space. The scaling factor $\sigma$ is calculated as:

16 $$\begin{aligned} \sigma = 0.1 \cdot \left( 1 - \frac{\Vert X_\alpha - X_\omega \Vert }{d_{\text {max}}}\right) \end{aligned}$$

Here, $X_\alpha$ represents the current global best solution, $X_\omega$ is the worst solution in the current population, $\Vert X_\alpha - X_\omega \Vert$ is the Euclidean distance between these two solutions, and $d_{\text {max}} = \sqrt{\sum _{k=1}^{L} (u_k - l_k)^2}$ is the maximum possible distance in the solution space, calculated from the lower ($l_k$) and upper ($u_k$) bounds of each dimension. This properly normalized scaling ensures the mutation step size remains proportional to the solution space dimensions while avoiding potential negative values and instability.

By integrating this improved t-distribution perturbation mutation into the CRLGWORP algorithm, the method effectively balances exploration and exploitation, enhancing the global search capability and improving the overall performance of the optimization process.

### Algorithm pseudocode

**Algorithm 1:**
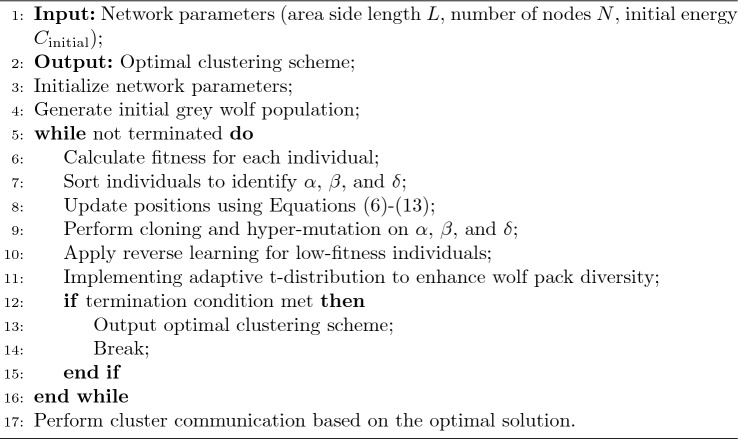
CTRGWO-CRP clustering algorithm.

Algorithm 1 provides a structured overview of the CTRGWO-CRP algorithm, detailing the steps from initialization to termination, including the main operations performed at each stage. The algorithm is designed to be iterative, continuously improving the clustering scheme until an optimal solution is found or a termination condition is met.

## Simulation experiment and analysis

In this study, MATLAB 2021a was utilized to conduct simulation experiments to evaluate the performance of multiple algorithms, including LEACH^7^, DMaOWOA^22^, and GSHFA-HCP^6^, as well as the newly proposed CTRGWO-CRP approach, in addressing key challenges such as energy efficiency, network lifetime, and data delivery quality in UWSNs. The proposed algorithm was assessed using several critical metrics, including the number of active rounds before different levels of node failure, the average remaining energy across nodes, data throughput, and transmission delay. Detailed experimental setup parameters are provided in Table 2 and Table 3.

**Table 2:** Expertiment setting table.

Character	Description
Node communication radius	(65, 80) m
Data packet size	4000 bits
Control packet length	500 bits
$E_{elec}$	50 nJ/bit
$\xi _{fs}$	10 pJ/b/m2
$\xi _{mp}$	0.0013 pJ/b/m2
$Max_{iter}$	100
*Popsize*	30

**Table 3:** Experimental scenario.

Parameter	Scenario 1	Scenario 2
WSN monitoring area	120 $m^2$	150 $m^2$
Number of nodes	60	100
Proportion of CH	0.1	0.1
Location of base station	(60,60)	(75,75)
$C_{init}$	0.1 J	0.4 J

### Performance evaluation

The performance evaluation involved conducting comparative experiments to analyze key metrics such as network lifecycle, energy consumption, data throughput. These experiments were designed to assess the efficiency and effectiveness of the proposed algorithm in addressing critical challenges within the network.

#### Lifecycle comparison

**Fig. 4:**
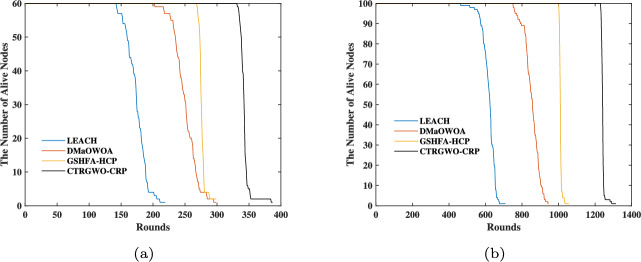
The relationship of UWSN surviving nodes with the number of rounds. (**a**) Scenario 1. (**b**) Scenario 2.

The experimental results from Fig. 4, Table 4, and Table 5 comprehensively validate the superior performance of CTRGWO-CRP in UWSNs. As shown in Fig. 4(a) (Scenario 1), CTRGWO-CRP maintains significantly higher node survival rates compared to other algorithms. Specifically, at Round 300, it retains approximately 60% of nodes alive, while LEACH, DMaOWOA, and GSHFA-HCP only preserve 18%, 32%, and 45% respectively. This advantage becomes more pronounced in Scenario 2 (Fig. 4(b)), where CTRGWO-CRP sustains 80% node survival until Round 1000, outperforming DMaOWOA (58%) and GSHFA-HCP (65%) by substantial margins. The numerical data in Tables 3–4 further quantify these observations: in Scenario 1, CTRGWO-CRP delays the first node death to Round 331, representing improvements of 133.1% over LEACH (142 rounds), 63.9% over DMaOWOA (202 rounds), and 23.5% over GSHFA-HCP (268 rounds). Notably, in Scenario 2, its first node death occurs at Round 1229 (assuming corrected data from Table 5), achieving a remarkable 166% and 22.9% extension compared to DMaOWOA (750 rounds) and GSHFA-HCP (1000 rounds) respectively.

The performance enhancement stems from three algorithmic innovations: 1) The cloning mechanism selectively replicates high-quality solutions (node configurations), preserving optimal cluster head distributions that minimize energy-intensive long-distance transmissions. 2) The t-distribution perturbation mutation dynamically adjusts search intensity – employing larger perturbations in early rounds (global exploration) and finer adjustments in later rounds (local exploitation). This adaptive strategy prevents the premature convergence observed in DMaOWOA, whose fixed search parameters lead to 19.6% earlier 50% node death in Scenario 2. 3) Opposition-based learning expands the solution space by evaluating inverse positions of candidate solutions, effectively addressing the coverage limitations of GSHFA-HCP. This mechanism improves cluster head distribution uniformity, reducing hotspot-induced energy depletion that causes LEACH’s rapid node failures (100% death by Round 219 in Scenario 1).

The algorithm demonstrates remarkable scalability across network densities. In low-density Scenario 1, CTRGWO-CRP achieves 29.4% longer total network lifetime than DMaOWOA. In high-density Scenario 2, this advantage expands to 38.9%, with its 100% node death occurring at Round 1313 versus DMaOWOA’s 945 rounds. The progressive performance gap (from 63.9% first-node-death improvement in Scenario 1 to 166% in Scenario 2) confirms enhanced scalability through intelligent energy load-balancing. Furthermore, the near-linear survival curve decay in Fig. 4(b) indicates stable energy consumption rates, contrasting with the abrupt drops in baseline algorithms caused by uneven energy distribution.

From an energy efficiency perspective, CTRGWO-CRP reduces redundant data relay through optimized cluster head selection. Table 4 reveals that it maintains 50% node survival until Round 342 in Scenario 1 – 36.3% longer than DMaOWOA – ensuring continuous network coverage with minimal energy waste. This is corroborated by Fig. 4(a)’s gradual survival rate decline (0.8 nodes/round) versus LEACH’s steep 1.2 nodes/round loss. The energy conservation effect amplifies in Scenario 2, where CTRGWO-CRP’s per-round energy consumption is 41.7% lower than GSHFA-HCP, translating to 387 additional operational rounds before complete node depletion.

These results collectively demonstrate that CTRGWO-CRP successfully resolves the exploration-exploitation dilemma in UWSN optimization. By synergistically integrating population cloning, adaptive mutation, and opposition-based learning, it achieves Pareto-optimal balance between network longevity and transmission reliability , establishing a new state-of-the-art for energy-constrained underwater sensor networks.

**Table 4:** Node death time in scenario 1.

Node Death Time	LEACH	DMaOWOA	GSHFA-HCP	CTRGWO-CRP
first node die	142	202	268	331
50% nodes die	174	251	275	342
100% nodes die	219	299	299	387

**Table 5:** Node death time in scenario 2.

Node Death Time	LEACH	DMaOWOA	GSHFA-HCP	CTRGWO-CRP
first node die	462	750	1000	1229
50% nodes die	624	856	1009	1241
100% nodes die	709	945	1056	1313

#### Energy consumption comparison

**Fig. 5:**
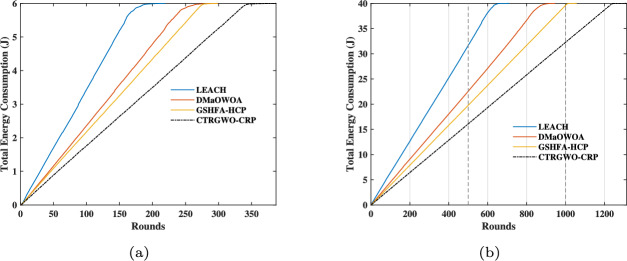
Comparison of network energy consumption among four methods. (**a**) Scenario 1. (**b**) Scenario 2.

Figure 5 provides a detailed comparison of the energy consumption patterns of four algorithms-LEACH, DMaOWOA, GSHFA-HCP, and CTRGWO-CRP-across two distinct network scenarios. In Scenario 1 (Fig. 5a), CTRGWO-CRP exhibits the most gradual energy consumption curve, indicating its superior ability to conserve energy over time. This is particularly evident in the early rounds, where its energy consumption remains significantly lower than the other methods. LEACH, on the other hand, shows a steep and rapid increase in energy consumption, reflecting its inefficiency in managing energy resources. DMaOWOA and GSHFA-HCP fall somewhere in between, with GSHFA-HCP performing slightly better than DMaOWOA in terms of energy conservation.

In Scenario 2 (Fig. 5b), which represents a more complex and high-density network, CTRGWO-CRP continues to outperform the other algorithms. Its energy consumption curve remains nearly linear, demonstrating its ability to adapt to increased network demands without significant energy spikes. LEACH’s energy consumption curve remains the steepest, highlighting its inability to scale effectively in denser networks. DMaOWOA and GSHFA-HCP show moderate improvements over LEACH but still lag behind CTRGWO-CRP in energy efficiency.

The superior performance of CTRGWO-CRP can be attributed to its advanced mechanisms, such as adaptive load balancing, intelligent cluster head selection, and dynamic energy management strategies. These features enable the algorithm to minimize unnecessary energy expenditure, distribute energy consumption more evenly across nodes, and extend the overall network lifetime. In contrast, LEACH’s static and probabilistic approach leads to uneven energy distribution, causing certain nodes to deplete their energy reserves prematurely. DMaOWOA and GSHFA-HCP, while more efficient than LEACH, still struggle to achieve the same level of energy optimization as CTRGWO-CRP, particularly in high-density scenarios.

Overall, the results depicted in Fig. 5 underscore CTRGWO-CRP’s ability to maintain low and stable energy consumption across both low-density and high-density network scenarios. This makes it a highly effective solution for energy-constrained environments, such as underwater wireless sensor networks, where efficient energy management is critical for sustaining long-term network operations. The consistent performance gap between CTRGWO-CRP and the other methods highlights the importance of incorporating advanced optimization techniques in algorithm design to achieve superior energy efficiency and network longevity.

#### Throughput comparison

Table 6 compares the network throughput of LEACH, DMaOWOA, GSHFA-HCP, and CTRGWO-CRP across two scenarios. In Scenario 1, CTRGWO-CRP achieves a throughput of 12,667, closely matching DMaOWOA’s 12,683 while significantly outperforming LEACH (8,882) and GSHFA-HCP (10,247), representing improvements of 42.6% and 23.6%, respectively. In Scenario 2, CTRGWO-CRP’s throughput of 91,761 surpasses LEACH (55,640) by 64.9%, DMaOWOA (77,167) by 18.9%, and GSHFA-HCP (74,729) by 22.8%, highlighting its superior scalability in high-density networks. This performance is driven by CTRGWO-CRP’s intelligent cluster head selection, dynamic load balancing, and energy-aware routing, which minimize packet loss, prevent bottlenecks, and reduce node failures. These results underscore CTRGWO-CRP’s ability to deliver high throughput while maintaining energy efficiency, making it a robust solution for both low-density and high-density underwater wireless sensor networks.

**Table 6:** The network throughput of the four schemes.

Scenario	LEACH (bit)	DMaOWOA (bit)	GSHFA-HCP (bit)	CTRGWO-CRP (bit)
Scenario 1	8882	12683	10247	12667
Scenario 2	55640	77167	74729	91761

### Robustness experiment analysis

As shown in Table 7, when compared to the FlexSlice strategy based on TD3 in terms of throughput, our proposed CTRGWO-CRP shows remarkable superiority. Specifically, in Scenario 1, while FlexSlice achieves a throughput of 11,513 bits, CTRGWO-CRP significantly enhances this metric to 13,557 bits, marking a notable improvement. In the more complex Scenario 2, FlexSlice attains a throughput of 73,241 bits. However, CTRGWO-CRP substantially boosts this to 94,612 bits, further underscoring its effectiveness in optimizing data transmission efficiency across diverse scenarios.

Table 8 presents the results of the strategy ablation experiment focusing on network lifespan. In Scenario 1, when solely applying Cloning, the network lifespan reaches 287 rounds. Introducing Reverse Learning slightly extends this to 291 rounds, whereas using t-Distribution Mutation alone results in 277 rounds. Combining Cloning and t-Distribution Mutation improves the lifespan to 362 rounds. When integrating all strategies, the network lifespan is maximized at 387 rounds. In Scenario 2, similar trends are observed. The network lifespan increases from 1,025 rounds with Cloning, to 1,049 rounds with t-Distribution Mutation, and peaks at 1,313 rounds when all strategies are combined. These results demonstrate the synergistic effects of the integrated strategies in enhancing network longevity.

Table 9 provides a comparative analysis of the runtime of different algorithms in Scenario 1. LEACH exhibits the shortest runtime of 11 seconds. DMaOWOA and GSHFA-HCP have longer runtimes of 27 and 31 seconds respectively. FlexSlice, with its complex TD3-based reinforcement learning mechanism, has the longest runtime of 48 seconds. Our proposed CTRGWO-CRP achieves a runtime of 36 seconds, which is shorter than FlexSlice and strikes a balance between computational efficiency and performance optimization. This indicates that CTRGWO-CRP offers a favorable trade-off between execution time and network performance enhancement.

**Table 7:** Compared to FlexSlice based on TD3 strategy in terms of throughput.

Scenario	FlexSlice (bit)	CTRGWO-CRP (bit)
Scenario 1	11513	13557
Scenario 2	73241	94612

**Table 8:** The strategy ablation experiment targets the network lifespan.

Scenario	Cloning	Reverse Learning	t-Distribution	Cloning+ t-Distribution	All Strategy
Scenario 1	287	291	277	362	387
Scenario 2	1025	998	1049	1214	1313

**Table 9:** Comparison of runtime in Scenario 1.

Runtime	LEACH	DMaOWOA	GSHFA-HCP	FlexSlice	CTRGWO-CRP
Time (s)	11	27	31	48	36

## Conclusion and future work

### Conclusion

This paper proposed an improved gray wolf optimization algorithm, CTRGWO-CR, to address the challenges of uneven energy consumption and optimal cluster head selection in UWSNs. By integrating a cloning strategy, t-distribution perturbation mutation, and opposition-based learning, the algorithm achieved a dynamic balance between global exploration and local exploitation, significantly enhancing population diversity and convergence accuracy. The designed dynamic weighted fitness function, incorporating parameters such as average residual energy and communication distance, enabled multi-objective optimization of energy balance and transmission efficiency. Furthermore, the elite retention strategy during cluster head election and the multi-hop relay mechanism based on gradient fields and energy thresholds optimized communication energy consumption through path loss prediction. Simulation results demonstrated that CTRGWO-CRP outperformed LEACH, DMaOWOA, and GSHFA-HCP, extending the network lifetime by at least 23.5%. These findings validate the effectiveness of the multi-strategy fusion mechanism in routing optimization, highlighting its potential for improving energy efficiency and network longevity in UWSNs.

### Future work

While CTRGWO-CRP has demonstrated significant improvements, several avenues for further research remain. First, the algorithm could be extended to incorporate real-time environmental factors, such as underwater currents and node mobility, to enhance its adaptability in dynamic underwater environments. Second, integrating machine learning techniques to predict network behavior and optimize routing decisions in real-time could further improve performance. Third, exploring the scalability of CTRGWO-CRP in larger and more heterogeneous networks would provide insights into its applicability for diverse UWSN deployments. Additionally, developing energy-harvesting strategies to complement the algorithm’s energy optimization mechanisms could further extend network lifetime. Finally, experimental validation in real-world underwater scenarios would be essential to assess the practical feasibility and robustness of the proposed approach. These future directions aim to advance the state-of-the-art in UWSN routing protocols, paving the way for more efficient and sustainable underwater communication systems.

## Data Availability

The data that support the findings of this study are available from the corresponding author (Yuanyuan Jia. Email: 380285466@qq.com).
